# Beyond the Blue: A Case Report on Diagnostic and Therapeutic Challenges in Infantile Methemoglobinemia With Incidental Genetic Mutation and Thrombotic Complication

**DOI:** 10.7759/cureus.91090

**Published:** 2025-08-27

**Authors:** Sania Shahid, Mariam Noor, Saif M Khan, Nusrath MP, Maryam Alsada

**Affiliations:** 1 Pediatric Emergency Medicine, Al Jalila Children's Hospital, Dubai, ARE; 2 Medicine, Tbilisi State Medical University, Tbilisi, GEO

**Keywords:** cerebral venous sinus thrombosis (cvst), congenital methemoglobinemia, emergency, methylene blue, methylene blue treatment, neonatal cyanosis, pediatric cyanosis, pediatric intensive care unit (picu)

## Abstract

Cyanosis in early infancy that is unresponsive to oxygen therapy is a life-threatening presentation requiring prompt diagnosis and intervention. While most cases are attributed to cardiopulmonary causes, hematologic disorders such as methemoglobinemia, although rare, remain important differentials. This report describes an eight-week-old male infant presenting with profound hypoxia, severe metabolic acidosis, and cyanosis. Methemoglobinemia was confirmed with markedly elevated levels. In the absence of oxidant exposure and a normal glucose-6-phosphate dehydrogenase (G6PD) screen, methylene blue was administered empirically with rapid clinical improvement. Rapid genomic sequencing revealed no pathogenic variants causative of methemoglobinemia. The case was further complicated by incidental venous sinus thrombosis and a transiently positive direct antiglobulin test. This case highlights the diagnostic and therapeutic challenges of methemoglobinemia in early infancy without a clear etiology, the cautious use of methylene blue pending enzymatic confirmation, and the interpretative limitations of rapid genetic testing in emergent settings.

## Introduction

Cyanosis in the pediatric age group typically signifies respiratory or cardiac pathology, yet when unresponsive to oxygen therapy, alternative hematologic or metabolic causes must be considered [1,2]. Methemoglobinemia is a rare but potentially fatal condition, occurring when hemoglobin iron is oxidized to the ferric state, rendering it incapable of binding oxygen [1,3]. It presents classically as central cyanosis that does not respond to oxygen, a saturation gap on arterial blood gases, and chocolate-brown blood [1,4]. Although acquired forms due to oxidant drugs, nitrates, or infection predominate in infants, congenital methemoglobinemia from cytochrome b5 reductase deficiency or hemoglobin M variants represents a rare but serious etiology [2,5]. Methylene blue remains the antidote of choice but is contraindicated in glucose-6-phosphate dehydrogenase (G6PD) deficiency due to the risk of hemolysis [1,3,6]. In the current era, rapid genomic sequencing offers promise in elucidating genetic causes of undifferentiated pediatric emergencies, although its limitations are increasingly evident in time-critical decisions [7-9].

## Case presentation

An eight-week-old term male infant, delivered via spontaneous vaginal delivery with no complications or neonatal intensive care unit (NICU) admission, presented to the emergency department with a one-day history of fever, two episodes of non-bilious, non-bloody vomiting, diarrhea, and decreased activity. The infant was exclusively formula-fed, with no exposure to medications or oxidants. On arrival, he appeared ill, cyanotic, and irritable, with oxygen saturations persistently between 25% and 80% despite supplemental oxygen and bag-valve-mask ventilation, although without overt respiratory distress. On detailed physical examination, the child was found to have tachycardia, with normal blood pressure, and was afebrile. Cardiovascular examination revealed no added heart sounds or murmurs. Respiratory assessment demonstrated no crackles or crepitations. Abdominal examination revealed gaseous distention with active bowel sounds, and the remainder of the systemic examination was unremarkable.

Arterial blood gas analysis demonstrated severe metabolic acidosis with a pH of 7.26, bicarbonate of 13.9 mmol/L, and lactate of 2.1 mmol/L. Methemoglobin level was markedly elevated at 63.8% (reference range: 0.5%-1.5%).

Initial laboratory investigations (Table 1) showed a hemoglobin of 9.6 g/dL, a white blood cell count of 34.9 × 10³/µL with lymphocytic predominance, and a positive direct antiglobulin test with C3b/C3d positivity.

**Table 1:** Key laboratory values on admission MPV: mean platelet volume, PCT: procalcitonin, G6PD: glucose-6-phosphate dehydrogenase, PT: prothrombin time, APTT: activated partial thromboplastin time, DAT: direct antiglobulin test

Laboratory test	Patient value	Reference range
Hemoglobin	9.6 g/dL	11-16 g/dL
White blood cell count	34.9 × 10³/µL	5-20 × 10³/µL
Platelet count	708 × 10³/µL (high)	210-500 × 10³/µL
MPV	9 fL	7.4-10.4 fL
Neutrophil (absolute)	9.42 × 10³/µL (high)	3-9 × 10³/µL
Lymphocytes (absolute)	19.20 × 10³/µL (high)	3-16 × 10³/µL
Monocytes (absolute)	5.24 × 10³/µL (high)	0.30-1.00 × 10³/µL
Eosinophils (absolute)	0.35 × 10³/µL	0.20-1.00 × 10³/µL
Peripheral blood film	Leucoerythroblastic changes	Normal morphology
C-reactive protein	23.2 mg/L (high)	0-5 mg/L
PCT	0.33 ng/mL	<0.5 ng/mL
Methemoglobin	63.8%	0.5%-1.5%
G6PD enzyme level	459 U/10¹²	146-376 U/10¹²
Total bilirubin	1.27 mg/dL	<1.2 mg/dL
PT	15.3 seconds	11.5-13.5 seconds
APTT	46.7 seconds	35.1-46.3 seconds
Dimer test	2.14 ug/mL	<0.5 ug/mL
DAT	Positive (C3b/C3d +1)	Negative
Creatinine	0.29 mg/dL (low)	0.31-0.53 mg/dL
Urea	17 mg/dL	7.276-35.952 mg/dL
Urinalysis (protein)	1	Negative
Urinalysis (red blood cells)	3-5 per high-power field	0-2 per high-power field
Peripheral blood film	Leucoerythroblastic changes	Normal morphology

A mild hyperbilirubinemia of 1.27 mg/dL and leucoerythroblastic changes on peripheral smear were noted. The G6PD screen was normal, although the quantitative assay showed an elevated enzyme level at 459 U/10¹² RBC (reference: 146-376 U/10¹²), raising concerns for a stress response or post-transfusion effect. Infectious workup, including blood and urine cultures, was negative, although stool polymerase chain reaction was positive for enteroaggregative *Escherichia coli*. In view of life-threatening hypoxia, methylene blue (2 mg/kg) was administered after qualitative G6PD clearance, as a normal G6PD level is an indication before methylene blue administration. Within six hours, methemoglobin levels declined to 2.2% with corresponding clinical improvement. These serial changes in acid-base balance and oxygenation before and after methylene blue therapy are summarized in Table 2.

**Table 2:** Serial arterial blood gas and methemoglobin values before and after methylene blue administration

Parameter	On arrival	Post-treatment (6 hours)	Reference range
pH	7.26	7.38	7.35-7.45
Bicarbonate	13.9 mmol/L	22.1 mmol/L	22-26 mmol/L
Lactate	8.1 mmol/L	1.8 mmol/L	0.5-2.0 mmol/L
Methemoglobin (%)	63.8%	2.2%	0.5%-1.5%
Oxygen saturation (%)	25%-80% (on oxygen)	>95% (on room air)	>95%

**Figure 1:**
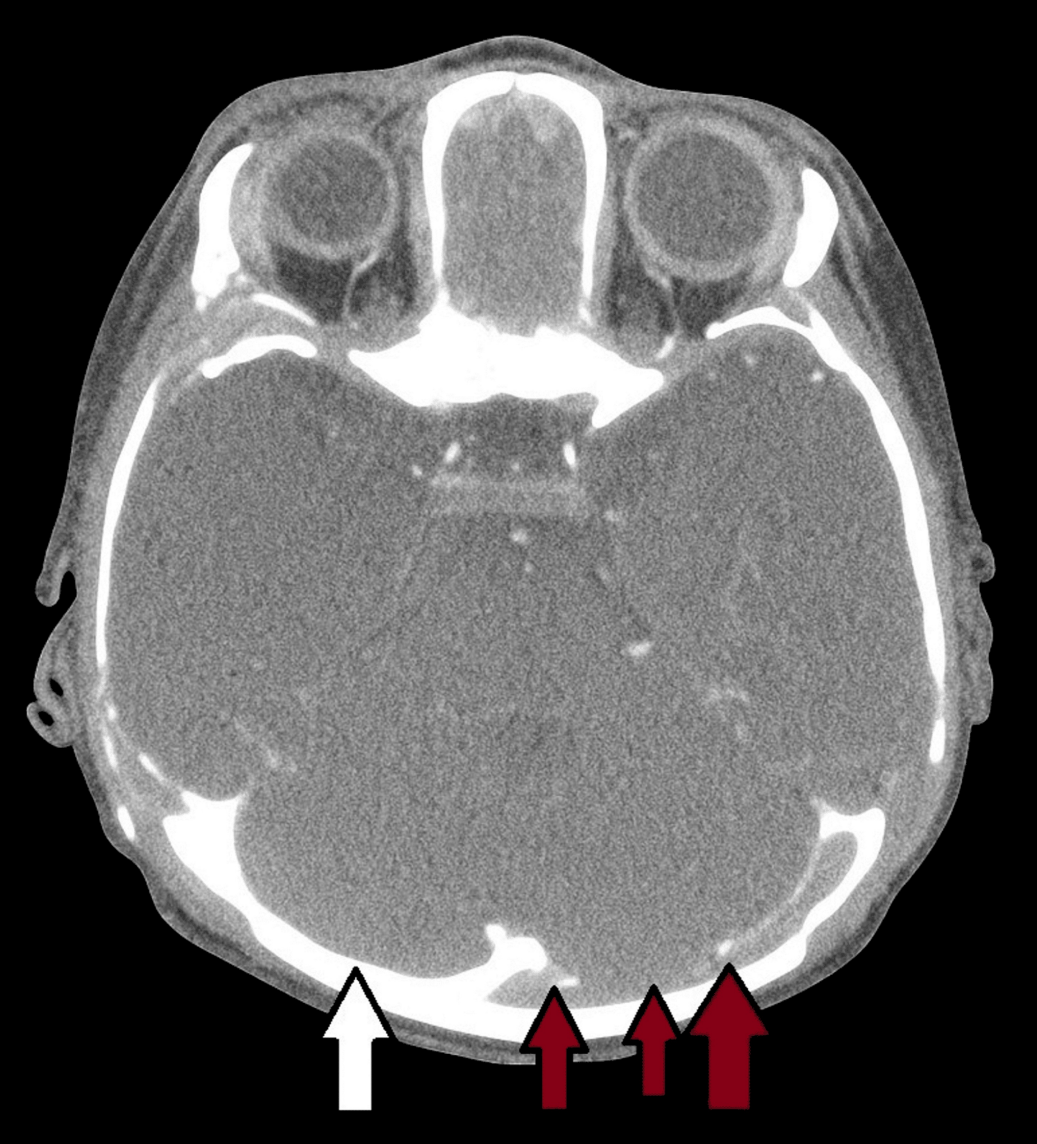
Brain CT with contrast showing partial central venous sinus thrombosis involving the superior sagittal and the left transverse sinus Red arrow showing filling defect within the posterior distal superior sagittal sinus with extension into the proximal left transverse sinus, representing a tiny residual thrombus. White arrow showing normal right transverse sinus pointed for comparison.

The patient's subsequent course was complicated by transient hypernatremia, metabolic acidosis, and anemia necessitating packed red blood cell transfusion. An incidental murmur on examination prompted echocardiography, revealing a small patent foramen ovale. Neurological imaging identified a partial superior sagittal and left transverse venous sinus thrombosis (Figure 1), for which therapeutic enoxaparin was initiated and titrated based on anti-Xa levels. Rapid genomic sequencing was performed to identify potential congenital causes, yielding a heterozygous pathogenic variant in BCS1L (p.Glu133Aspfs*25), associated with autosomal recessive mitochondrial complex deficiency, but not implicated in methemoglobinemia.

No pathogenic or likely pathogenic variants related to methemoglobinemia were detected. Despite incidental findings, the patient demonstrated gradual normalization of laboratory parameters, resolution of cyanosis, and satisfactory weight gain on extensively hydrolyzed formula. The infant was discharged on enoxaparin for planned six-week therapy and outpatient hematology and neurology follow-up.

**Figure 2:**
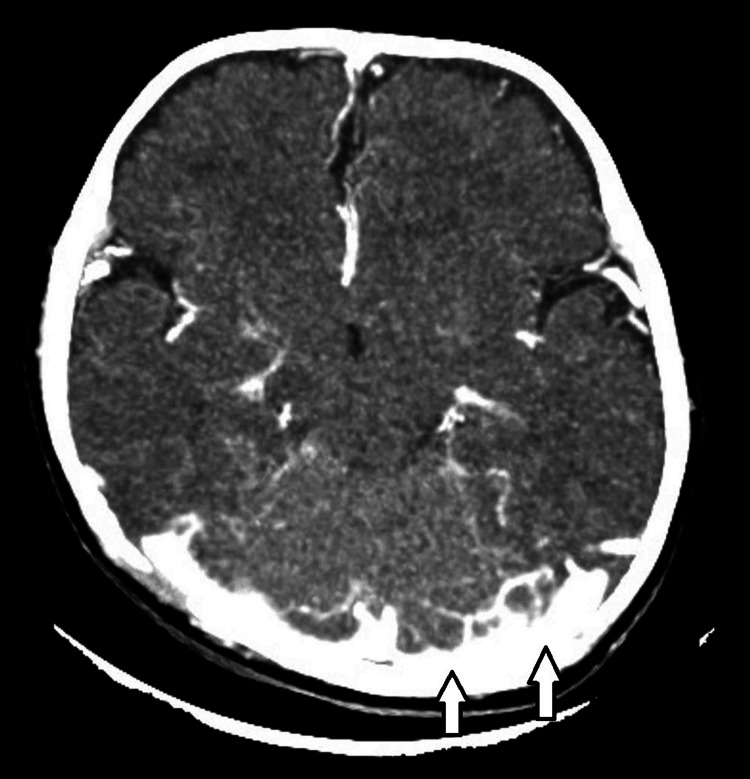
Follow-up brain CT scan showing resolution of the left-sided filling defect (normal left superior sagittal and the left transverse sinus)

Repeat CT done upon follow-up showed resolution of the thrombus, as shown in Figure 2.

## Discussion

Methemoglobinemia in pediatrics remains a rare but critical diagnosis, often presenting as unexplained cyanosis resistant to supplemental oxygen [1-3]. The condition results from increased methemoglobin formation or impaired reduction to hemoglobin via the cytochrome b5 reductase pathway [2,3]. While most cases are acquired due to oxidizing agents or sepsis, congenital forms, although exceedingly rare, must be excluded when no exogenous cause is identified [1,4,5]. Severe cases can present with saturations below 85%, refractory to oxygen, alongside metabolic acidosis and shock [4,5].

In this patient, the absence of known oxidant exposure and the early age of onset raised suspicion for a congenital etiology. Methylene blue remains first-line therapy, acting as an artificial electron carrier in the NADPH-methemoglobin reductase pathway. However, its use mandates exclusion of G6PD deficiency due to the risk of precipitating acute hemolysis. Although the qualitative screen was normal, the elevated quantitative G6PD level complicated interpretation, likely reflecting a reactive marrow response, active hemolysis, or technical artifact. Literature suggests that even in G6PD-deficient patients, methylene blue may be cautiously considered when life-threatening hypoxia is present, provided risks are communicated and monitored, as in this case [1-3].

The detection of enteroaggregative *E. coli* may have contributed to stress erythropoiesis and hemolysis, reflected in the blood film and positive direct antiglobulin test. Although rare, hemolytic anemia and methemoglobinemia have been associated with sepsis in neonates [4,5]. The transient venous sinus thrombosis likely resulted from dehydration, hypernatremia, and hemoconcentration, a recognized complication in critically ill infants [6]. Rapid genomic sequencing was pursued, given the severity and unusual presentation. While valuable for diagnosing undifferentiated illness, sequencing yields actionable results in a minority of acute cases [7-9]. The incidental heterozygous BCS1L mutation was of uncertain relevance, as biallelic variants are typically required for disease manifestation.

Methemoglobinemia and venous sinus thrombosis are rarely reported together; a potential pathophysiological connection may exist. Methemoglobinemia reduces the oxygen-carrying capacity of blood, leading to tissue hypoxia. Hypoxia, in turn, is known to promote endothelial dysfunction and activate hypoxia-inducible factors, which can enhance coagulation pathways and predispose to thrombosis [6]. Additionally, altered nitric oxide signaling in the presence of elevated methemoglobin may contribute to vascular dysregulation and a prothrombotic milieu. These mechanisms may provide a plausible explanation for the development of venous sinus thrombosis in the context of methemoglobinemia, although further evidence is needed [6]. In addition, a thrombophilia and coagulation disorder screen, including testing for common inherited and acquired thrombophilias, was performed and found to be normal for our patient. This supports an acquired etiology for the venous sinus thrombosis, likely related to dehydration and hypernatremia, rather than an underlying prothrombotic disorder.

Previous reports highlight the importance of maintaining a high index of suspicion for methemoglobinemia in infants with unexplained cyanosis. Cases with similar presentations but without identified genetic causes have been described, emphasizing empirical management [10-12]. This case also underscores the diagnostic limitations of current rapid genomic platforms in the acute setting, where clinical judgment often supersedes genetic findings [13,14]. The utility of methemoglobin-specific co-oximetry for rapid diagnosis, prompt administration of methylene blue, and coordinated multidisciplinary care were crucial to this patient's outcome.

## Conclusions

This case illustrates the critical importance of considering methemoglobinemia in a cyanotic infant unresponsive to oxygen therapy. Prompt empirical administration of methylene blue can be lifesaving, even in the absence of immediate enzymatic or genetic confirmation. While rapid genomic sequencing can support etiological diagnosis, its limitations in acute pediatric care necessitate reliance on clinical acumen. In this case, the venous sinus thrombosis required initiation of anticoagulation with close follow-up, including serial CT imaging; therapy was discontinued once resolution was confirmed, underscoring the importance of integrating thrombotic complications into both acute management and long-term care planning.

Incidental findings, such as a heterozygous BCS1L mutation and venous sinus thrombosis, underscore the complex interplay between critical illness, diagnostic uncertainty, and the challenges of patient management. This case highlights the importance of comprehensive, multidisciplinary care, involving pediatric intensivists, neurologists, hematologists, cardiologists, geneticists, radiologists, pharmacologists, and specialized nursing staff, to ensure timely diagnosis, targeted interventions, and optimized patient outcomes.
